# Data-Driven Prediction of the Formation of Co-Amorphous Systems

**DOI:** 10.3390/pharmaceutics15020347

**Published:** 2023-01-20

**Authors:** Elisabeth Fink, Michael Brunsteiner, Stefan Mitsche, Hartmuth Schröttner, Amrit Paudel, Sarah Zellnitz-Neugebauer

**Affiliations:** 1 Research Center Pharmaceutical Engineering, Inffeldgasse 13, 8010 Graz, Austria; 2 Celeris Therapeutics GmbH, Salzamtsgasse 7, 8010 Graz, Austria; 3 Institute of Electron Microscopy and Nanoanalysis (FELMI), Graz University of Technology, Steyrergasse 17, 8010 Graz, Austria; 4 Graz Centre for Electron Microscopy (ZFE), Steyrergasse 17, 8010 Graz, Austria; 5 Institute for Process and Particle Engineering, Graz University of Technology, Inffeldgasse 13, 8010 Graz, Austria

**Keywords:** machine learning, gradient boosting, co-amorphous, molecular descriptors, inhalation therapy

## Abstract

Co-amorphous systems (COAMS) have raised increasing interest in the pharmaceutical industry, since they combine the increased solubility and/or faster dissolution of amorphous forms with the stability of crystalline forms. However, the choice of the co-former is critical for the formation of a COAMS. While some models exist to predict the potential formation of COAMS, they often focus on a limited group of compounds. Here, four classes of combinations of an active pharmaceutical ingredient (API) with (1) another API, (2) an amino acid, (3) an organic acid, or (4) another substance were considered. A model using gradient boosting methods was developed to predict the successful formation of COAMS for all four classes. The model was tested on data not seen during training and predicted 15 out of 19 examples correctly. In addition, the model was used to screen for new COAMS in binary systems of two APIs for inhalation therapy, as diseases such as tuberculosis, asthma, and COPD usually require complex multidrug-therapy. Three of these new API-API combinations were selected for experimental testing and co-processed via milling. The experiments confirmed the predictions of the model in all three cases. This data-driven model will facilitate and expedite the screening phase for new binary COAMS.

## 1. Introduction

Most of the marketed inhalation products target local lung diseases, such as chronic obstructive pulmonary disease (COPD) and asthma. Another important area for pulmonary drug delivery is the treatment of tuberculosis. In all these cases, a multidrug-therapy applying different active pharmaceutical ingredients (APIs) is necessary, which still depends on the administration of multiple different commercial products. Administering an inhalable glucocorticoid (IGC), together with either a long-acting beta-adrenoreceptor agonist (LABA) or a long-acting muscarinic receptor antagonist (LAMA), has become the mainstay of long-term therapy to relieve symptoms of asthma as well as COPD [1]. On the other hand, combinations of short-acting beta adrenoreceptor agonists (SABA) and short-acting muscarinic receptor antagonists (SAMA) are used as quick-relief medications. They are commonly administered in acute asthma attacks and show a stronger bronchodilator response than single APIs [2]. The treatment of tuberculosis is usually more complex and requires a multidrug regime therapy administered over a long period [3]. In the initial intensive phase, a combination of first-line drugs is required for at least two months. In the continuation phase, a combination of two or three drugs is used for at least four months to sterilize lesions in the lung. So far, anti-tuberculosis drugs that are administered via the oral route, or parenterally, have been designed. Meanwhile, inhalable anti-tuberculosis drug formulations have also gained considerable research interest, since they offer direct delivery of the API to the local therapeutic target in the respiratory tract [4,5,6,7].

Therefore, co-processed systems comprised of two APIs (used in treatment of the afore mentioned diseases) are suggested in order to reduce dose variability and enhance patient compliance, by reducing the number of different medications to take, as well as leaner and more economic manufacturing of the same. Such formulations will provide processing benefits as they are produced in a single step and thus unit operations, for example mixing of different APIs, becomes dispensable. For solid oral dosage forms, crystalline products are often preferred due to their stability. However, co-crystals have a fixed stoichiometry and the resulting dose-ratios may not comply with clinical requirements [8]. In this work we propose co-amorphous systems (COAMS) that combine improved stability properties with a wider range of possible dose ratios [9].

Generally, COAMS have been described as a promising solution to stabilize amorphous forms and combine their advantages (e.g., increased solubility, faster dissolution rates, and potentially higher bioavailability) with the stability of crystalline forms [10]. COAMS are defined as homogenous single-phase systems comprised of two or more molecules, stabilized predominantly through intermolecular interactions. Typically, an API is combined with a low molecular weight compound, the so-called co-former, which can be either another API or an excipient. Depending on the co-former used, COAMS can be divided into several classes: (a) amino acid based COAMS, (b) drug based COAMS, (c) organic acid based COAMS, and (d) other COAMS [11]. Experimental methods to obtain COAMS include co-melting or melt quenching [12], solvent evaporation [13], spray-drying [14] or freeze-drying [15], and milling [16].

An appropriate co-former selection is crucial for successful co-formability. However, a lack of systematic, predictive, and computational methods for co-former selection has been identified [17]. So far, co-former selection has mainly been done on a case-by-case basis, based on synergistic effects and/or combination therapy, previous studies, physicochemical properties, or structural analysis. Approaches include the prediction of the miscibility of two compounds involving (a) Hansen Solubility parameters [18], (b) Flory–Huggins parameter [12], (c) co-former selection based on physicochemical properties of the co-former like glass transition temperature, melting point and molecular flexibility [18], or (d) certain other molecular descriptors [19,20]. More details and a good overview of the existing literature on the rational selection of co-formers can be found in the recent review by Yarlagadda et al. [21].

There is continued need for a predictive model that is able to identify potential COAMS (involving two compounds) in a time- and cost-effective screening process. The aim is to be able to make predictions about the hypothetical formation of a COAMS via the calculation of the molecular descriptors even before the synthesis of a new active ingredient. To date, the abilities of predictive screening tools for COAMS formations are limited, since they focus on a restricted set of compounds only. For example, the predictive identification of co-formers was studied for five APIs, with the selection of co-formers limited to amino acids [11,19]. Based on Partial Least Square analysis-discriminant analysis (PLS-DA), a predictive model was built and validated with a single API (mebendazol). The overall accuracy, being the percentage of correct predictions of the model, was 81% [11]. In order to build a more general model for predicting COAMS, we created a dataset containing information on combinations of two substances already described in the literature (including all classes mentioned earlier) to be used as training data for a machine learning (ML) approach.

ML has revolutionized the processing of data in many industries by enabling data-driven predictions [22]. By calibrating a large number of independent parameters, ML attempts to recognize patterns in data and to map underlying relationships in the form of mathematical functions. Generally, ML techniques can be split into two categories, being either supervised or unsupervised [23]. The first category requires labelled training data, such as for example annotated images or data records with an assigned category, as in the present case with COAMS and non-COAMS. Supervised ML includes methods such as decision trees or neural networks. Unsupervised ML detects patterns in datasets without requiring prior knowledge about the data and includes methods such as clustering.

The review articles by Lou et al., and Nagy et al. provide a good overview on the application of ML tools in solid oral dosage forms [24], and the application of artificial neural networks (ANN) including ML in pharmaceutical manufacturing [25]. Some specific examples where ML tools were applied in the pharmaceutical industry are process monitoring and control of hot-melt extrusion [26], understanding tablet properties [27], the prediction of co-crystal formation [28], and understanding the parameter space for the calibration of simulations [29]. Machine learning models have also been widely used in the last few years to predict properties of molecules based on their structure. In particular, in drug discovery such methods have been shown to be very successful [30]. Numerical values describing the molecular structure have also been used to predict for example sweetness [31] or bitterness [32]. Other examples include the prediction of the formation of co-crystals [33].

In our case, a decision tree based model, using a gradient boosting classifier from the Python package XGBoost [34], was developed to predict the successful formation of COAMS. In order to avoid bias, a cross-validation approach was used and 50 models were trained independently after hyper-parameter-tuning. The predictions from the 50 created models were then averaged to obtain a final value for the prediction. The resulting data-driven model was used for computationally identifying API-API co-particle formulation systems, with the ultimate goal of better therapeutic prospects for asthma/COPD or tuberculosis patients (through enhancing patient compliance by reducing the number of medications to take). In addition, this approach could help to make manufacturing simpler and leaner.

A predictive ML model for COAMS formation would not only be beneficial for inhalation therapy in the screening phase, to save time and cost, but also in other pharmaceutical fields, for example for identifying co-formers for promising new but poorly soluble drugs. The range of values within which the application of the model is trustworthy is shaped by the underlying data. The general workflow for constructing the predictive ML model, such as selection of the ML tool, the hyper-parameter-tuning, and the split of data into training and test data, would remain mostly the same on similar, but novel data. Hence, we foresee that the model could be easily adapted to other application areas by changing or expanding the underlying data.

## 2. Materials and Methods

### 2.1. Data Collection

#### 2.1.1. Composition of Data

Data of COAMS reported in the literature was the basis for developing the ML model. This included information on pharmaceutical co-amorphous systems described in the literature, as well as systems that were tested for co-amorphization, but that did not form a co-amorphous system. Besides the identity of the two compounds and the information on COAMS formation (COAMS or non-COAMS), also their molar ratio and the preparation technique (either via a thermodynamic or kinetic pathway [35]) were added. Additional data on the stability and properties of the formed COAMS were added, if available. However, the choice of parameters studied was not uniform among the different research reports, and the stability experiments performed in each case differed, for example, in terms of temperature or humidity settings. Consequently, data on stability was used for cleansing purposes but not for model building. COAMS for which no stability data were available were removed from the list. Further, data curation was performed on systems that were listed multiple times and, dependent on the molar ratio and/or the preparation technique, either listed as COAMS or non-COAMS. For example, if one system was reported several times as COAMS and only in one molar ratio, and/or with a certain preparation technique non-COAMS. These subtle differences were not expected to be learned sufficiently well by any model with the amount of training data available. Hence, they were deemed inconsistencies and removed. Only with a larger available dataset is it possible to make these more in-depth differentiations. Additionally, systems which were tested repeatedly in various molar ratios, and with different techniques, were kept in the list a maximum of three times. The most common molar ratios were 1:1, 1:2 and 2:1. These steps were undertaken to ensure the diversity of the training data in order to obtain a valid, generalisable model.

We divided the data into four classes based on the combination of the API with (1) another API, (2) an amino acid, (3) an organic acid, or (4) another substance. Our database now lists COAMS from all four classes. After data cleaning and curation, the database contained 244 systems (Supplementary Material Table S1). On the basis of individual compounds, this resulted in 47 APIs and 40 co-formers.

#### 2.1.2. Training and Test Data

The collected data was split into training data and test data. The test data was decided to account for 1/12 of the data (19 rows). In order to determine this, random splits were performed and a suitable, representative selection of test data was chosen. The criterion for this was that examples of all four classes, and both non-COAMS and COAMS, should be included in a similar proportion as in the training data. The training data was used for the ML model building and hence composed of 225 systems: 69 class-1 systems, 90 class-2 systems, 46 class-3 systems, and 20 systems from class-4. Of these, 154 are COAMS and 71 are non-COAMS.

The test data, which was not included in the training data for model building, was composed of 12 positive (COAMS) and 7 negative (non-COAMS) examples from 13 APIs and 15 co-formers.

### 2.2. Descriptor Selection

The canonical SMILES (simplified molecular-input line-entry system) strings of all compounds from the training data and the test data were collected from PubChem^®^ (National Library of Medicine [36], Bethesda, MD, USA). The SMILES is a specification in the form of a line notation for describing the structure of chemical species. With the Python package MORDRED (a molecular-descriptor calculation package [37]), over 1800 available molecular descriptors were determined for each compound. The number of calculated descriptors exceeds the number of training data by an order of magnitude. This is generally not recommended, because as a consequence the parameters of the model cannot be trained sufficiently. Hence, the number of molecular descriptors was reduced to 29, based on previous models in COAMS modelling [11,18,19] and mechanistic considerations (regarding mechanisms involved in COAMS stabilisation like, e.g., the number of hydrogen bond donor/acceptors or the acidic/basic group count). Alternatively, an automatic selection could have been performed, which chooses the descriptors based on their predictive power on the data. However, it would remain unclear whether the descriptors selected in this way would actually be representative or simply work best with the available data. Hence, an approach based on a literature review and mechanistic considerations was chosen instead. The selected descriptors are listed in Table 1. These molecular descriptors served as input factors for the ML model.

For each pair of substances (forming either COAMS or non-COAMS), the absolute value of the difference between the API and co-former values of the molecular descriptors was calculated. This resulted in 29 input features. Another four input features were calculated to describe hydrogen bonds and acid/base interactions between the systems. Here, the relationship of donors and acceptors is more relevant. Consequently, respective parameters were calculated based on methods from Chambers et al. [11]. These four additional input features are: nAB (absolute difference of nAcid of substance 1 and nBase of substance 2), nBA (absolute difference of nBase of substance 1 and nAcid of substance 2), nHBDA (absolute difference of nHBDon of substance 1 and nHBAcc of substance 2), nHBAD (absolute difference of nHBAcc of substance 1 and nHBDon of substance 2). Further, the preparation technique (thermodynamic or kinetic) was added as an input feature. In total, this resulted in 34 input features.

### 2.3. Modelling Tool

Generally, ML methods are statistical tools for approximating the dependency of one or several output variables on one or several input variables. One well-known example is linear regression. In order to also capture non-linear dependencies, most ML techniques typically use a combination of mathematical functions, e.g., [22]. In order to validate and test a model, the available data is usually split into training, validation, and test data. The training data is used to calibrate the available parameters of the approximation function equation, whereas the validation data is used during training to, for example, avoid over-fitting. The test data is used after training to evaluate the performance and generalisability of a model on unseen data. In order to avoid the observed accuracy of a model being only due to a fortunate choice of the validation data, cross-validation is performed. For any type of cross-validation, the part of the training data kept for validation is typically varied and the average accuracy for a model type is noted.

In this model, as a first step the molecular descriptors named in Section 2.2 (Table 1) were calculated for each entry in the data (training and test data) obtained from the literature. This resulted in a table containing both substances for forming the COAMS or non-COAMS as well as the molecular descriptors for each of them. As a target output value for the prediction, a binary classification into non-COAMS (0) and COAMS (1) was used. Several ML methods are suitable for this task of binary classification. In our case, due to the small size of the training data, a pre-selection was made after in-depth analysis of the underlying mathematical concepts [38]. Popular ML methods include for example, random forest (RF) [39], extreme gradient boosting (XGB) [40], k-nearest-neighbors (KNN) [41], support vector machine (SVM) [42], and artificial neural networks (ANN) [43]. The first two methods are based on a combination of decision trees. The underlying concept is a sequence of binary trees, where each node in each tree represents a yes/no decision [44]. This makes them scale invariant and due to the relatively small number of parameters that need to be determined, they are well-suited for small datasets. The third method, KNN, is a graph-based approach working best with uniformly scaled data, but it also produces satisfactory results when only a small amount of data is available. The very popular ANN is an application of iterated matrix multiplications, with typically non-linear functions applied in each step. For the approximation of functions on many variables, as in this case where we have 34 input factors, very large weight matrices would have to be trained. Hence, this method is in general not well-suited for small datasets and was therefore not considered.

**Figure 1:**
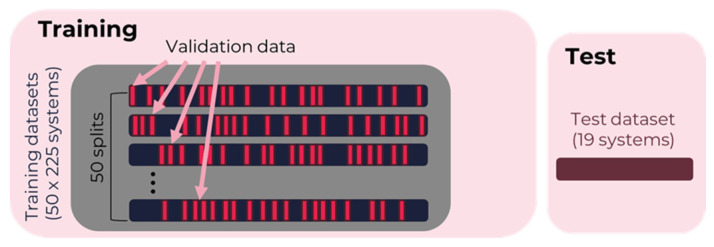
Split of training data with 50-fold cross-validation and additional test data. The selection of the validation data is performed randomly each time.

Based on the preference to tune a small number of parameters during the training, the following methods were selected for investigation: RF (Python, scikit-learn 0.24.2), XGB (Python, XGBoost 1.4.2), SVM (scikit-learn 0.24.2), and KNN (Python, scikit-learn 0.24.2). There are several different ways to cross-validate a model. One common method is k-fold cross-validation, which is performed by using 10–20% of the data as the validation data, where these 10–20% are shifted across the data so that the validation data for all training runs are pairwise disjoint sets. In particular, for smaller datasets the two models with disjoint validation, and hence training data, can be quite different in their performance due to a lucky or unlucky choice of this dataset split. To avoid such a bias, and in order to obtain a more balanced, statistically representative model, here a different form of cross-validation was chosen. It can be easily tested that the resulting model in tree-based methods such as XGB depends on the order of the rows within the dataset. This effect is particularly visible when the size of the dataset is relatively small. The order of the data in this approach was randomly shuffled before each run, and 15% of the training data were randomly selected as validation data for this individual model. This method of cross-validation is described and compared in [45]. Here, the predictions of the models from 50-training runs, with a random 85%/15% split for training and individual validation data, were averaged for all three ML methods. Figure 1 illustrates the split of training data for each training run into the training and validation parts, as well as the completely separate unseen test data (an additional 19 systems) for testing the performance of the resulting combined model.

For each ML technique, hyper-parameter tuning was performed via a grid search, where all possible combinations of hyper-parameter values were tested if there was more than one parameter. Table 2 shows an overview of the methods and hyper-parameter ranges. For each ML method, training was performed with all pairwise combinations of values for the hyper-parameters.

The performance of the tested ML methods was evaluated based on the training accuracy as well as the validation accuracy. The accuracy was determined based on the percentage of correct predictions.

### 2.4. Application

In many cases, the treatment of lung diseases is based on a combination of pairs of APIs. Predicting the formation of COAMS is therefore an ideal application of the developed ML model. Consequently, 35 common APIs used in the treatment of asthma, COPD, and tuberculosis [46,47] were identified. A complete list of the APIs considered can be found in Supplementary Material (Tables S2 and S3).

For all these APIs, the previously selected molecular descriptors (see Section 2.1.1) were calculated based on the canonical SMILES of the APIs. For testing the generated ML model to predict co-amorphous API-API combinations out of these APIs for inhalation therapy, all possible API-API combinations were selected and the difference of the respective molecular descriptors calculated and used as input features. The molar ratio was set at 1:1 and the preparation method as either thermodynamic or kinetic. This resulted in a list of 666 new combinations in total.

### 2.5. Distance from Training Data—Uncertainty Factor

The applicability of the generated ML model to predict the formation of COAMS in novel combinations of substances is restricted by their similarity to the training data used. Results for API combinations whose molecular descriptors deviate significantly from those of the training data are therefore less reliable. Consequently, the distance of all new combinations to the training data was calculated by using the Euclidean distance between these two 34-dimensional vectors, which corresponds to the length of the path between them. This distance was then considered in addition to the predicted score when evaluating new systems. Further, a high value for the predicted score was desired since this reflects a higher certainty of the model prediction. Accordingly, promising new COAMS are selected based on a high predicted score (close to 1) and a short distance from the training data. An uncertainty factor (UF)for positive predictions (*p* values ≥ 0.5), combining the predicted score and distance from the training data was introduced as:

Equation (1): Definition of the uncertainty factor (UF).

(1) $$UF\left(x\right)=\frac{d\left(x,T\right)}{p{\left(x\right)}^{4}}$$

The formula in Equation (1) to obtain the *UF* was determined so that it scales predictions based on their distance from the training data. In order to achieve this, the Euclidean distance was divided by the fourth power of the predicted value. The UF was calculated for all new systems with a predicted score of 0.5 or higher (a large probability to form COAMS). Here, d(x,T) is the distance of a new combination x from the training data T, and p(x) is the predicted score ranging from 0.5 to 1. The fourth power of the predicted score in the denominator has the effect that systems with a predicted value for *p* greater than but close to 0.5 are given a very high UF. Such predictions are then considered highly doubtful and are questionable when selecting systems for experimental testing. The factor UF was calculated for all API-API combinations considering a thermodynamic as well as a kinetic preparation method. Since results were comparable, those for kinetic methods only are shown.

### 2.6. Experimental Model Validation

#### 2.6.1. Materials

Experimental ML model validation was done on three examples, two positively predicted COAMS and one negatively predicted COAMS. The selection was determined by the availability of APIs intended for the inhalation therapy in-house. Consequently, glycopyrronium bromide (GB, Chiesi Pharmaceutics, Parma, Italy), budesonide (BUD, TCI Deutschland GmbH, Eschborn, Germany), ethambutol (ETH, TCI Deutschland GmbH, Eschborn, Germany), and streptomycin sulphate (STR, TCI Deutschland GmbH, Eschbor, Germany) were used.

#### 2.6.2. Milling Expriments

For initial experimental model validation, milling, a common kinetic method described for co-amorphization, was chosen. One gram mixtures of the two selected APIs were pre-weighed in a molar ratio of 1:1. A list of the three API combinations, the weights used, the predicted values and the distances from the training data are reported in Table 3.

The APIs were weighed on an analytical balance (XP205DR, Mettler Toledo, Leicester, UK). Then the mixtures were placed in a 50 mL mixing cylinder together with a stainless-steel ball (20 mm diameter). Afterwards, the cylinder was placed in a Retsch cryomill (Retsch GmbH, Haan, Germany) operated without nitrogen. Milling was done at 25 Hz for 10 min. After 5 min the mill was stopped and a 5 min break was made to avoid overheating and degradation of the APIs. After a second milling cycle of 5 min, samples were stored in a desiccator.

#### 2.6.3. X-ray Powder Diffraction (XRPD) Analysis

The individual APIs (before milling) and the milled API-API blends were immediately analysed via XRPD (maximum time from milling to analysis was around 1 h). The analysis was performed with a Siemens D5005 (in Bragg-Brentano geometry equipped with a Cu-Anode (λ = 1.54186 A) operated at 40 kV and 40 mA. The measurements were performed in a 2 Theta range between 4° and 40° with a step size of 0.04° and a time per step of 2 s. A scintillator detector was used for counting the X-rays.

## 3. Results and Discussion

### 3.1. Model Performance—Accuracy

Based on the training data, 50 individual models were built for all ML techniques and hyper-parameter combinations from Table 2. For the predictive model, the average of those 50 predictions was used. Table 4 summarizes the performance of each tested method on all data and separately on the validation part. The performance of each method was evaluated in Table 4 with the respectively best hyper-parameter choice.

**Figure 2:**
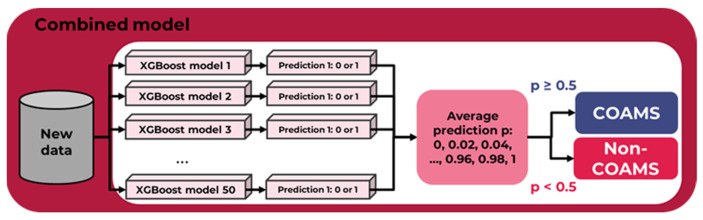
Outline of the combined overall ML model when making predictions for new data.

As can be seen in Table 4, except for SVM all methods are quite similar in their performance. Since KNN is highly scale sensitive, for training all input features’ value ranges are scaled to be mapped into the interval [0, 1] to obtain some uniformity. This, however, could lead to skewed results in the generalisation. Hence, a tree-based method was chosen. As such, gradient boosting is less dependent on a fortunate hyper-parameter choice, and was selected as the modelling tool, with 18 estimators and a maximum depth of 6. We averaged the predictions of 50 models (each of them predicting 1 or 0 for COAMS or non-COAMS, respectively), resulting in a higher overall accuracy. This increases the model’s overall applicability to new data, as required for future application. If at least half of the individual ML models output the value 1 (i.e., from a threshold value of 0.5), the combined overall ML model will predict the successful formation of a COAMS (see Figure 2). The value p reflects the probability for forming a COAMS. If the value is very close to one, then it is very likely that a system is COAMS. However, if the value is equal to, or only slightly greater than, 0.5, the model will predict a system to be COAMS but with a low certainty. This dynamic was considered when applying the model to new systems.

**Figure 3:**
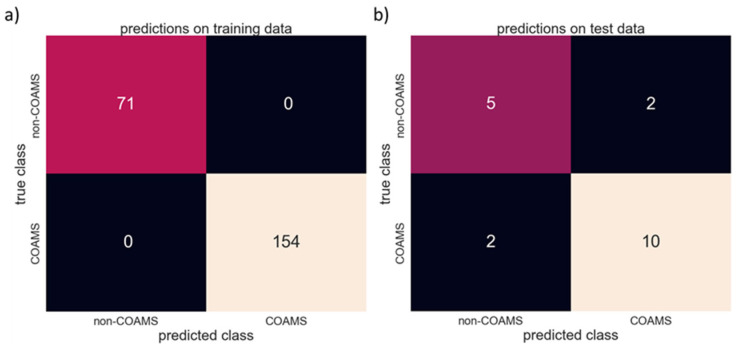
Model accuracy on the (**a**) training data and (**b**) test data.

On average, the individual XGBoost classifier models achieved an accuracy of 97%. When combined, the overall model then achieved an accuracy of 100% on the training data. In more detail, 70 out of 71 non-COAMS samples, and all 157 COAMS samples, were predicted correctly. Figure 3a summarizes the accuracy of the combination of 50 XGBoost classifier models on the training data. However, since every system in the dataset will have very likely been part of the training data in most of these 50 models, this is neither surprising nor an indicator for high overall accuracy. Hence, for more reliable information on the performance of this model, it is necessary to evaluate the accuracy with additional data that was never used in the training. When using the test data (see Section 2.1.2) as input for the model, this resulted in an accuracy of 79%. Based on 19 additional systems (12 COAMS, 7 non-COAMS), 10 out of 12 COAMS and five out of seven non-COAMS were identified correctly, while two non-COAMS were falsely predicted to be co-amorphous. Figure 3b summarizes the accuracy achieved during testing.

### 3.2. Relevance of Molecular Descriptors

Black box models are generally not desirable, because a comprehensible computational path is a prerequisite for the credibility of its conclusions. Several predictive modelling techniques offer the possibility to extract information about the importance of each input factor after training. For example, multivariate analysis of co-former selection for naproxen identified crystallization tendency, glass transition temperature (Tg), and molecular flexibility as relevant factors [18]. In this case, physicochemical parameters were either determined experimentally (e.g., Tg from DSC experiments) or via in silico calculations (e.g., logP, aromatic ring number or topological polar surface area (TPSA)), using different programs, for example ACD/Percepta (Advanced Chemistry Development Inc., Toronto, Canada) or Molecular Operating Environment (Ryoka Systems Inc., Tokayo, Japan)). Other earlier models investigated selected systems from a single class of COAMS only by calculating descriptors via COSMOquick, a program calculating, among other things, the interaction parameters between two compounds. In that case, a large value of molecular weight, the sum of the difference between hydrogen bond donors and acceptors for both substances, a relatively small value for excess enthalpy of mixing, excess energy of hydrogen bonding, and the difference in the Hansen Solubility parameter for the co-former and the API [11] were found favorable for the formation of COAMS.

The priority for the present modelling approach was for the input parameters (SMILES combined with selected MORDRED descriptors) to be easily accessible, and to generate a comprehensible and reliable model to predict the formation of COAMS across all four classes. For the latter, the identification of the most critical individual chemical parameters of each substance involved is highly complex. In COAMS, to stabilize the amorphous form, mechanisms range from intermolecular interactions like hydrogen-bonding and ionic interactions (salt formation), to non-functional interactions (e.g., molecular mixing). Accordingly, which mechanism finally drives the formation of a selected COAMS depends on the individual partners and thus on the class [17,48].

Tree-based models, such as extreme gradient boosting, allow easy extraction of critical features from the final model, providing detailed understanding of the relative contribution of each input factor to the model’s predictive decisions. In XGBoost, the importance is calculated for each single decision tree by the amount that each attribute node improves the prediction, weighted by the number of observations that this node has been used for [49]. The overall score for the importance of each feature is then averaged over all trees in the model. If all input factors are contributing equally to the prediction, the relative importance of each is simply 1/n, where n is the number of input factors. However, the relative importance of an individual input factor actually depends on the composition and order of data within the training data. For example, if a single parameter varies little within the training data, then it may consequently be underestimated in the prediction. Indeed, it is possible that this exact parameter has a central role for the formation of a COAMS in a system that has not been covered so far. Therefore, a large heterogeneity in the training data is the basis for the validity of the model.

**Figure 4:**
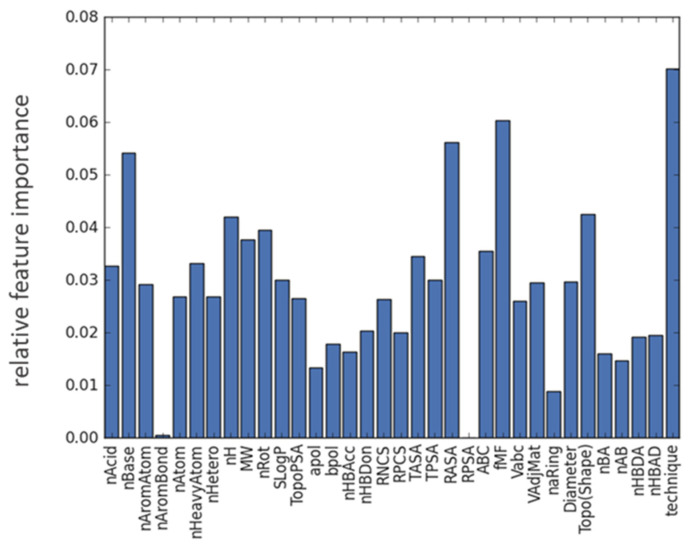
Feature importance plot highlighting the relative contribution of each descriptor (see Table 1) to the overall prediction averaged over ten training runs.

Additionally, the trees in an XGBoost classifier are built based on the order of the data within the dataset. In relatively small datasets, such as the present one, it is not uncommon to observe a strong dependency of the calculated feature importance on the order of the data. Meaning, if the data is shuffled, in the sense that rows are randomly re-arranged, the importance of individual features can, under certain circumstances, be rather different. Since we have 34 input features, the threshold for deeming a factor important is 1/34 = 0.029, with higher values indicating more significance of the corresponding factor to the final prediction. In order to obtain reliable and stable indicators for feature importance, 10 repetitions of training the entire model, each comprised of 50 XGBoost classifier models, were undertaken. Within these, the feature importance from each repetition was evaluated by using the method feature_importances_ from XGBoost classifier. In all repetitions, the order of the impact of each factor remained the same, although the absolute values varied slightly, as is to be expected. Based on our training data, for our predictive ML model the following parameters were identified to be mainly contributing to COAMS formation (Figure 4): RASA (0.056 ± 0.015), fmF (0.055 ± 0.025), nBase (0.053 ± 0.019), nH (0.047 ± 0.019), nRot (0.45 ± 0.025), Topo (Shape) (0.043 ± 0.015), and preparation technique (0.066 ± 0.038) (mean ± SD from 10 evaluations).

For all input features, the average value for COAMS and non-COAMS was compared. If a feature, which is the difference in absolute values for this input factor, was significantly smaller in COAMS than in non-COAMS, it was concluded that high similarity in this molecular descriptor was beneficial for the formation of COAMS. For example, a small value of the difference in relative hydrophobic surface area (RASA) promotes the formation of COAMS in comparison to non-COAMS. So, if this parameter is more similar for two compounds, the formation of a COAMS is more likely. This also applies for the molecular framework ratio (fmF) and the topological shape index (Topo (Shape)). Whereas, all formed COAMS showed a larger value for the difference of nRot, nH and nBase. So, to form a COAMS, two substances should preferably differ in these parameters. fmF [50] and Topo(Shape) are shape descriptors of the molecule and related to the appearance of a substance, and it seems that the more similar two substances are in this regard the more likely it is that a COAMS is formed.

The rotable bond count (nRot) gives the number of bonds which allow free rotation around themselves. These are defined as any single bond, not in a ring, bound to a non-terminal heavy atom, excluding amide C–N bonds due to their high rotational energy barrier [51]. Substances with high nRot, are assumed to be flexible in orientating and if two substances are diverse in this parameter they are more likely to approach, and to interact, with each other.

It is not surprising that the preparation technique has been shown to play a major role in the prediction of COAMS. The model has learned from training data where certain systems work with one technique only. Selecting an appropriate preparation technique has been shown to be crucial to achieving a satisfactory performance of the final products. Usually, properties of the two substances, e.g., drug and co-former, both influence the selection of the preparation method [10]. For example, heat sensitive substances cannot be processed via melt-quenching, or certain substances have been shown to only form COAMS with a specific preparation technique such as carvedilol and amino acids, where a COAMS was formed after spray-drying, but ball milling of the same substances was not successful in co-amorphization [52].

Interestingly, parameters like nHDon and nHAcc, and their cross relations, were not deemed important, unlike in previous studies [11]. This is most likely due to the fact that, compared to these studies, where only amino acids were investigated as co-formers, we considered COAMS from four different classes. This involves more different co-former types besides amino acids and consequently also more variety in mechanisms involved in stabilizing the COAMS, so that overall this factor was not identified as being important in our study.

### 3.3. Modelling Performance—Application

**Figure 5:**
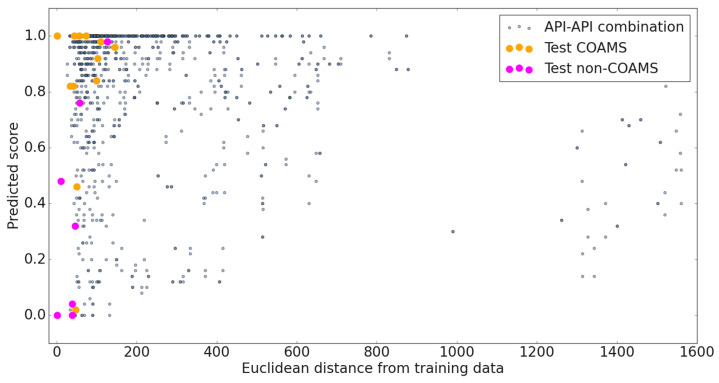
Graphical description of the predictions for the test data and all new API-API combinations. Vertically, the average of the predicted class (1 = COAMS, 0 = non-COAMS) from 50 mL models is depicted, horizontally, the distance from the original training data.

To test the suitability of the model for the field of inhalation therapy, 35 APIs used in the treatment of asthma, COPD and tuberculosis were selected. As mentioned, its applicability to predict the formation of COAMS in novel combinations of substances is restricted by their similarity to the training data used. Consequently, the UF (Equation (1)) considering precision and distance to the training data was introduced. A graphical description of the predictions for all new API-API combinations (blue dots), and their distance from the training data, is visualized in Figure 5 together with the test data. Combinations with a high score (close to 1 on the *y*-axis) are likely to form co-amorphous systems. The horizontal distance of the individual points from the *y*-axis provides information about the similarity of the new combination to the training data.

The UF values for all possible API-API systems considered ranged from 37 to 11,578. The lower the UF, the more accurate the prediction. The 100 combinations with the lowest UF were selected to be considered for future screening. This corresponds to those combinations with a UF below 77, likely forming COAMS.

As mentioned in the introduction, administering IGCs together with LABAs or LAMAs has become the mainstay of long-term therapy of asthma as well as COPD. Products on the market combining two individual APIs from these classes are, for example, salmeterol/fluticasone (Seretide^®^, GSK, Durham, NC, USA), formoterol/budesonide (Symbicort^®^, AstraZeneca, Cambridge, UK), beclomethasone/formoterol (Foster^®^, Chiesi, Parma, Italy), mometasone/formoterol (Dulera^®^, Merck, Germany), and fluticasone/formoterol (Flutiform^®^, Mundipharma, Frankfurt, Germany). Promising combinations identified by our model are mometasone—bambuterol (prediction 1.0, UF 57) or mometasone—glycopyrronium bromide (prediction 0.98, UF 58), and budesonide—tiotropium (prediction 0.98, UF 70) or budesonide—glycopyrronium bromide (prediction 0.98, UF 74). They yielded both a high predicted score and an acceptable UF. A combination of SABA and SAMA to be administered in acute asthma identified by our ML model is fenoterol—ipratropium (prediction 0.94, UF 74). A general trend of the COAMS formation for systems containing quaternary amine anti-cholinergic drugs with ICS and beta-blockers warrants further molecular level mechanistic investigation.

For the treatment of tuberculosis, respective first line drug combinations identified by our model are for example ethambutol—pyrazinamide (prediction 0.98, UF 47) and ethambutol—isoniazid (prediction 0.98, UF 59).

With regard to the predictability of the developed model, there is a new COAMS system recently described in the literature that was not included in our dataset, budesonide and theophylline [53]. The prediction of our generated ML model for this combination was 0.94 and the distance from the training data 71.9; a first indicator confirming the predictive power of the model. Experimental validation of the model was initially done for 3 systems (2 positively predicted and one negatively predicted API-API combination), and results are presented in the section below.

### 3.4. Experimental Model Validation

For the initial model validation, three relatively clear systems with respect to prediction and UF/distance were deliberately chosen. Further, selection criteria were the availability of APIs for inhalation therapy in-house, cost of the APIs, and having at least one therapeutic relevant system included. Based on these criteria, two systems with high predicted scores (1 and close to one) and a low and medium distance (74.2 and 411.0), and one example with a very low predicted score (0) and low distance (89.0) were selected. Table 5 lists the 3 API-API combinations that were co-processed via milling.

**Figure 6:**
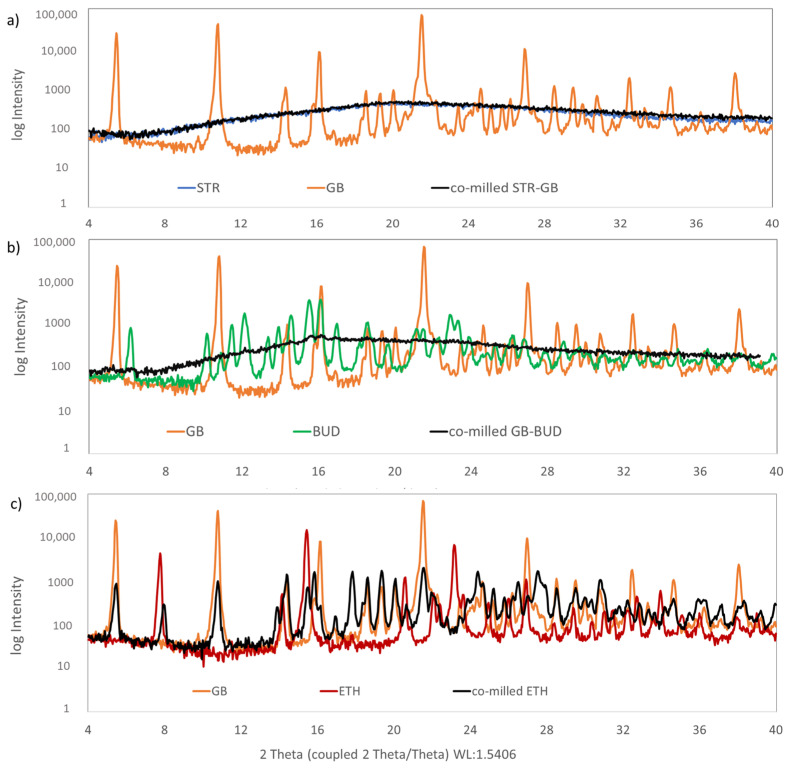
XRPD pattern of starting materials (API 1 and API 2) and co-milled samples of (**a**) streptomycin sulphate (STR) and glycopyrronium bromide (GB), (**b**) glycopyrronium bromide and budesonide (BUD) and (**c**) glycopyrronium bromide and ethambutol (ETH).

Figure 6 shows XRPD patterns of the starting materials and the three co-milled samples. Each of the graphs (Figure 6a–c) shows the two starting materials (API 1 and API 2) and the corresponding co-milled sample.

Except for streptomycin sulphate (STR), all starting materials (APIs) were crystalline, indicated by their characteristic diffraction pattern. STR as a starting material did not show any characteristic Bragg peaks, which is a clear indication of an amorphous material. Glycopyrronium bromide (GB) showed characteristic peaks at 2θ of 5.22°, 10.80°, 14.36°, 21.52° and 27.44 [54,55]. Budesonide (BUD) showed characteristic peaks at 2θ of 5.99°, 11.95°, 14.42°, 15.36° and 15.96° indicating its crystalline nature. These data were in agreement with previously published data [56]. Ethambutol (ETH) showed three intense characteristic crystalline peaks at 2θ of 7.86°, 15.48° and 23.25° [57].

Looking at the co-milled API-API combination STR-GB, the crystalline peaks of the GB starting material disappeared, and only a background signal was visible, indicating a co-amorphous system (Figure 6a). For co-milled BUD-GB, also the characteristic Bragg peaks of GB and BUD starting material disappeared, showing the typical background for an amorphous material (Figure 6b). By contrast, the co-milled GB-ETH sample still indicated characteristic peaks from GB as well as ETH starting materials (Figure 6c). This matches with the prediction from the developed ML model (Table 5).

## 4. Conclusions and Outlook

In this study, we used literature data to build a molecular descriptor-based ML model for predicting the potential of binary drug combinations to form COAMS. In contrast to previously reported predictive models, we used input data from four classes of COAMS (combinations of an API with (1) another API, (2) an amino acid, (3) an organic acid, or (4) another substance). The accuracy of the generated ML model was 79%. The applicability of the model was tested on 35 APIs used in the therapy of asthma, COPD, and tuberculosis with the goal of predicting promising co-amorphous API pairs for improved therapy.

A simple tool for a quick screening for the potential formation of co-amorphous systems was successfully created. With this, it is possible to reduce the experimental effort, and save time and cost. Further, the workflow to develop the model can easily be applied to other applications by changing the input data.

In a next step, the model will be further experimentally validated with the goal of identifying promising co-amorphous API-API combinations for inhalation therapy. Therefore, API-API combinations that are clinically relevant based on current therapy regimes, and that are close to the original training data (examples mentioned above), will be tested. Regarding clinical relevance, further tests about efficacy, safety, etc., are necessary, and will have to be considered in a later phase. In order to further optimize the model, and to determine the limits up to which the prediction is fairly reliable, additionally, carefully selected systems with a variety of precision and distance values will be tested. The selection of these systems will aim to improve the coverage of the data space. This will provide the basis for a fine-tuned ML model, which will then be able to more accurately predict a broader range of COAMS, not only for the therapy of lung disease.

## Figures and Tables

**Table 1:** Selected molecular descriptors and respective abbreviations.

ABC	Atom bond connectivity index	Diameter	Topological diameter	RNCS	Relative negative charge surface area
nAcid	Acidic group count	Topo-ShapeIndex	Topological shape index	RPCS	Relative positive charge surface area
nBase	Basic group count	nRot	Rotatable bonds count	TASA	Total hydrophobic surface area
nAromAtom	Aromatic atoms count	SLogP	Wildman-Crippen log P	TPSA	Total polar surface area
nAromBond	Aromatic bond count	TopoPSA	Topological polar surface area	RASA	Relative hydrophobic surface area
nAtom	Number of all atoms	naRing	Aromatic ring count	RPSA	Relative polar surface area
nHeavyAtom	Number of heavy atoms	apol	Atomic polarisability	fMF	Molecular framework ratio
nHetero	Number of hetero atoms	bpol	Bond polarisability	Vabc	ABC van der Waals volume
nH	Number of H atoms	nHBAcc	Number of hydrogen bond acceptor	VAdjMat	Vertex adjacency information
MW	Molecular weight	nHBDon	Number of hydrogen bond donors		

**Table 2:** Tested ML methods and hyper-parameters with ranges used during hyper-parameter-tuning.

ML Method	Hyper-Parameter	Description	Values
Random forest	N_estimators	Number of trees	[3,5,8,10,15]
XGBoost_classifier	N_estimators	Number of trees	[3,5,8,10,15]
	Max_depth	Depth of the individual trees	[2,3,5,7,10,12,15]
SVM			
KNN	k	Number of neighbours used for a prediction	[3,5,8,10,15]

**Table 3:** API combinations for the milling experiments. The APIs were combined in a molar ratio of 1:1. The respective weights are given in the Table.

API 1		API 2		Model Prediction	Distance from Training Data
budesonide (BUD)	574.84 mg	glycopyrronium bromide (GB)	425.16 mg	0.98 (COAMS)	74.2
glycopyrronium bromide (GB)	179.34 mg	streptomycin sulphate (STR)	821.02 mg	1 (COAMS)	411.0
ethambutol (ETH)	390.66 mg	glycopyrronium bromide (GB)	608.88 mg	0 (non-COAMS)	89.3

**Table 4:** Comparison of ML methods’ performance.

Data	KNN	SVM	XGBoost Classifier	Random Forest
Training data	97%	89%	97%	97%
Validation data	84%	83%	85%	85%

**Table 5:** List of API-API combinations for experimental model validation, the prediction, distance and XRPD result.

API 1	API 2	Prediction	Distance	XRPD Results
GB	STR	1	411.0	COAMS
BUD	GB	0.98	74.2	COAMS
ETH	GB	0	89.3	non-COAMS

## Data Availability

Not applicable.

## Supplementary Materials

The following supporting information can be downloaded at: https://www.mdpi.com/article/10.3390/pharmaceutics15020347/s1, Table S1. Results Literature Review (Data used for Model Building and Testing); Table S2. List of APIs used in the treatment of COPD and Asthma (Input for Model Application test); Table S3. List of APIs used in the treatment of Tuberculosis (Input for Model Application test).
