# Supplementary material for: Data-Driven Prediction of the Formation of Co-Amorphous Systems
Source: Pharmaceutics. 2023 Jan 20;15(2):347. doi: 10.3390/pharmaceutics15020347 (PMC9968185; doi:10.3390/pharmaceutics15020347)
Supplement: Supplementary file 1 [file pharmaceutics-15-00347-s001.zip › pharmaceutics-2141181-supplementary.pdf]

Supplementary Material

Table S1 – Results Literature Review (Data used for Model Building and Validation)

Class*	Substance 1	Substance 2	Molar Ratio	Technique	Technique classified **	COAMS	Reference
2	Indomethacin	Arginin	1:1	Vibrational ball milling	Kinetic	Yes	[1]
2	Indomethacin	Tyrosin	1:1	Vibrational ball milling	Kinetic	No	[1]
2	Indomethacin	Phenylalanin	1:1	Vibrational ball milling	Kinetic	Yes	[1]
2	Indomethacin	Tryptophan	1:1	Vibrational ball milling	Kinetic	Yes	[1]
2	Carbamazepine	Arginin	1:1	Vibrational ball milling	Kinetic	No	[1]
2	Carbamazepine	Tyrosin	1:1	Vibrational ball milling	Kinetic	No	[1]
2	Carbamazepine	Phenylalanin	1:1	Vibrational ball milling	Kinetic	No	[1]
2	Carbamazepine	Tryptophan	1:1	Vibrational ball milling	Kinetic	Yes	[1]
1	Indomethacin	Naproxen	1:1	Quench cooling	Thermodynamic	Yes	[2]
1	Simvastatin	Glipizide	2:1	Ball milling	Kinetic	Yes	[3]
1	Simvastatin	Glipizide	1:1	Ball milling	Kinetic	Yes	[3]
1	Simvastatin	Glipizide	1:2	Cryomilling	Kinetic	Yes	[3]
1	Lurasidone HCl	Repaglinide	2:1	Solvent evaporation	Thermodynamic	Yes	[4]
1	Lurasidone HCl	Repaglinide	1:1	Solvent evaporation	Thermodynamic	Yes	[4]
1	Lurasidone HCl	Repaglinide	1:2	Solvent evaporation	Thermodynamic	Yes	[4]
1	Naproxen	Cimetidine	2:1	Ball milling	Kinetic	Yes	[5]
1	Naproxen	Cimetidine	1:1	Ball milling	Kinetic	Yes	[5]
2	Budesonide	Arginin	1:1	Spray drying	Thermodynamic	Yes	[6]
3	Palbociclib	Succinic acid	1:1	Ball milling	Kinetic	Yes	[7]
3	Palbociclib	Tartaric acid	1:1	Ball milling	Kinetic	Yes	[7]
3	Palbociclib	Citric acid	1:1	Ball milling	Kinetic	Yes	[7]
3	Palbociclib	Malic acid	1:1	Ball milling	Kinetic	Yes	[7]
1	Nifedipine	Naproxen	1:1	Melting 120°	Thermodynamic	Yes	[8]
1	Nifedipine	Loratadine	1:1	Melting 120°	Thermodynamic	Yes	[8]
1	Nifedipine	Indomethacin	1:1	Melting 120°	Thermodynamic	Yes	[8]
1	Nifedipine	Itraconazole	1:1	Melting 120°	Thermodynamic	Yes	[8]
4	Nifedipine	Saccharin	1:1	Melting 120°	Thermodynamic	No	[8]
4	Nifedipine	D-Salicin	1:1	Melting 120°	Thermodynamic	Yes	[8]
4	Probucol	Sucrose	1:1	Melting 120°	Thermodynamic	No	[8]
1	Probucol	Indomethacin	1:1	Melting 120°	Thermodynamic	Yes	[8]
1	Probucol	Carvedilol	1:1	Melting 120°	Thermodynamic	No	[8]
1	Probucol	Cimetidine	1:1	Melting 120°	Thermodynamic	No	[8]
1	Itraconazole	Indomethacin	1:1	Melting 120°	Thermodynamic	Yes	[8]
1	Itraconazole	Clotrimazole	1:1	Melting 120°	Thermodynamic	Yes	[8]
1	Itraconazole	Cimetidine	1:1	Melting 120°	Thermodynamic	Yes	[8]
1	Itraconazole	Carvedilol	1:1	Melting 120°	Thermodynamic	Yes	[8]
4	Itraconazole	Maltitol	1:1	Melting 120°	Thermodynamic	No	[8]

4	Itraconazole	Sucrose	1:1	Melting 120°	Thermodynamic	No	[8]
1	Indomethacin	Felodipine	1:1	Melting 120°	Thermodynamic	Yes	[8]
1	Indomethacin	Felodipine	1:3	Melting 120°	Thermodynamic	Yes	[8]
1	Indomethacin	Cimetidine	1:1	Melting 120°	Thermodynamic	Yes	[8]
1	Indomethacin	Sucrose	1:1	Melting 120°	Thermodynamic	No	[8]
1	Clotrimazole	Carvedilol	1:1	Melting 120°	Thermodynamic	Yes	[8]
1	Cimetidine	Indomethacin		Solvent evaporation	Thermodynamic	Yes	[9]
1	Ritonavir	Indomethacin	2:1	Solvent evaporation	Thermodynamic	Yes	[10]
1	Ritonavir	Indomethacin	1:1	Solvent evaporation	Thermodynamic	Yes	[10]
1	Ritonavir	Indomethacin	1:2	Solvent evaporation	Thermodynamic	Yes	[10]
1	Atorvastatin calcium	Carvedilol	1:1	Solvent evaporation	Thermodynamic	Yes	[11]
1	Atorvastatin calcium	Glibenclamide	1:1	Solvent evaporation	Thermodynamic	Yes	[11]
4	Atorvastatin	Nicotinamide	1:1	Solvent evaporation	Thermodynamic	Yes	[11]
1	Ezetimibe	Indapamid	2:1	Quench cooling	Thermodynamic	Yes	[12]
1	Ezetimibe	Indapamid	1:1	Quench cooling	Thermodynamic	Yes	[12]
1	Ezetimibe	Indapamid	1:2	Quench cooling	Thermodynamic	Yes	[12]
1	Nateglinide	Metformin HCl	1:1	Ball milling	Kinetic	Yes	[13]
1	Nateglinide	Metformin HCl	1:3	Ball milling	Kinetic	Yes	[13]
1	Nateglinide	Metformin HCl	1:5	Ball milling	Kinetic	Yes	[13]
4	Talinolol	Naringin	1:1	Quench cooling	Thermodynamic	Yes	[14]
4	Talinolol	Naringin	1:2	Quench cooling	Thermodynamic	Yes	[14]
4	Talinolol	Naringin	2:1	Quench cooling	Thermodynamic	Yes	[14]
1	Glipizide	Atorvastatin	1:1	Cryomilling	Kinetic	Yes	[15]
1	Glipizide	Atorvastatin	1:2	Cryomilling	Kinetic	Yes	[15]
1	Glipizide	Atorvastatin	2:1	Cryomilling	Kinetic	Yes	[15]
1	Naproxen	Indomethacin		Spray drying	Thermodynamic	Yes	[16]
1	Omeprazole	Amoxicillin anhydrous	1:1	Co-grinding	Kinetic	Yes	[17]
1	Tranilast	Diphenhydramine HCl	2:1	Quench cooling	Thermodynamic	Yes	[18]
1	Tranilast	Diphenhydramine HCl	1:1	Quench cooling	Thermodynamic	Yes	[18]
1	Tranilast	Diphenhydramine HCl	1:2	Quench cooling	Thermodynamic	Yes	[18]
1	Indomethacin	Cimetidine	1:1	Quench cooling	Thermodynamic	Yes	[19]
1	Naproxen	Cimetidine	1:1	Quench cooling	Thermodynamic	Yes	[19]
1	Indomethacin	Cimetidine	1:1	Co-evaporation	Thermodynamic	Yes	[19]
1	Naproxen	Cimetidine	1:1	Co-evaporation	Thermodynamic	Yes	[19]
1	Indomethacin	Cimetidine	1:1	Ball milling	Kinetic	Yes	[19]
1	Naproxen	Cimetidine	1:1	Ball milling	Kinetic	Yes	[19]
4	Repaglinide	Saccharin	3:1	Solubility crystallization	Thermodynamic	Yes	[20]
4	Repaglinide	Saccharin	2:1	Solubility crystallization	Thermodynamic	Yes	[20]
4	Repaglinide	Saccharin	1:1	Solubility crystallization	Thermodynamic	Yes	[20]
4	Lurasidone HCl	Saccharin	1:1	Solvent evaporation	Thermodynamic	Yes	[21]

2	Naproxen	Tryptophan	1:1	Ball milling	Kinetic	No	[22]
2	Simvastatin	L-Aspartic acid	1:1	Cryomilling	Kinetic	No	[23]
2	Simvastatin	Lysine	1:1	Cryomilling	Kinetic	Yes	[23]
2	Simvastatin	Serine	1:1	Cryomilling	Kinetic	No	[23]
2	Simvastatin	Threonine	1:1	Cryomilling	Kinetic	No	[23]
2	Glibenclamide	L-Aspartic acid	1:1	Cryomilling	Kinetic	No	[23]
2	Glibenclamide	Lysine	1:1	Cryomilling	Kinetic	No	[23]
2	Glibenclamide	Serine	1:1	Cryomilling	Kinetic	Yes	[23]
2	Glibenclamide	Threonine	1:1	Cryomilling	Kinetic	Yes	[23]
2	Serine	Threonine	1:1	Cryomilling	Kinetic	No	[23]
2	Indomethacin	Arginin	1:1	Spray drying	Thermodynamic	Yes	[24]
2	Indomethacin	Arginin	1:1	Spray drying	Thermodynamic	Yes	[24]
2	Indomethacin	Tryptophan	1:1	Ball milling	Kinetic	Yes	[25]
2	Furosemide	Tryptophan	1:1	Ball milling	Kinetic	Yes	[25]
2	Simvastatin	Lysine	1:1	Spray drying	Thermodynamic	Yes	[26]
3	Paracetamol	Citric acid	1:3	Melt-quenching	Thermodynamic	Yes	[27]
3	Paracetamol	Citric acid	1:1	Melt-quenching	Thermodynamic	Yes	[27]
3	Paracetamol	Citric acid	3:1	Melt-quenching	Thermodynamic	Yes	[27]
3	Clozapine	Oxalic acid	1:1	Solvent evaporation	Thermodynamic	Yes	[28]
3	Clozapine	Oxalic acid	1:2	Solvent evaporation	Thermodynamic	Yes	[28]
3	Clozapine	Oxalic acid	1:1	Solvent evaporation	Thermodynamic	Yes	[28]
3	Clozapine	Oxalic acid	1:2	Solvent evaporation	Thermodynamic	Yes	[28]
3	Clozapine	Tartaric acid	1:1	Solvent evaporation	Thermodynamic	Yes	[28]
3	Clozapine	Tartaric acid	1:1.5	Solvent evaporation	Thermodynamic	Yes	[28]
3	Clozapine	Tartaric acid	1:2	Solvent evaporation	Thermodynamic	Yes	[28]
3	Azelnidipine	Maleic acid	1:1	Neat powder grinding	Kinetic	Yes	[29]
3	Azelnidipine	Maleic acid	1:2	Neat powder grinding	Kinetic	Yes	[29]
3	Acyclovir	Citric acid	1:2	Solvent evaporation	Thermodynamic	Yes	[30]
2	Valsartan	Histidine	1:1	Ball milling	Kinetic	Yes	[31]
2	Valsartan	Arginin	1:1	Ball milling	Kinetic	Yes	[31]
2	Valsartan	Lysine	1:1	Ball milling	Kinetic	Yes	[31]
1	Citric acid	Indomethacin	1:1	Co-mellting	Thermodynamic	Yes	[32]
3	Sulfamerazine	Deoxycholic acid	1:1	Cryomilling	Kinetic	Yes	[33]
3	Sulfamerazine	Citric acid	1:1	Cryomilling	Kinetic	Yes	[33]
3	Sulfamerazine	Sodium taurocholate	1:1	Cryomilling	Kinetic	Yes	[33]
3	Sulfathiazol	Glutaric acid	1:1	Milling	Kinetic	No	[34]
3	Sulfathiazol	Oxalic acid	1:1	Milling	Kinetic	No	[34]
3	Sulfathiazol	Tartaric acid	1:1	Milling	Kinetic	Yes	[34]
3	Sulfathiazol	Citric acid	1:1	Milling	Kinetic	Yes	[34]
3	Sulfathiazol	Adipic acid	1:1	Milling	Kinetic	No	[34]
3	Sulfathiazol	Pimelic acid	1:1	Milling	Kinetic	No	[34]
3	Sulfathiazol	Malonic acid	1:1	Milling	Kinetic	No	[34]
3	Sulfathiazol	Malic acid	1:1	Milling	Kinetic	No	[34]

3	Sulfathiazol	Succinic acid	1:1	Milling	Kinetic	No	[34]
3	Sulfathiazol	Fumaric acid	1:1	Milling	Kinetic	No	[34]
1	Naproxen	Cimetidine	1:1	Ball milling	Kinetic	Yes	[5]
1	Naproxen	Cimetidine	1:2	Ball milling	Kinetic	Yes	[5]
1	Naproxen	Cimetidine	2:1	Ball milling	Kinetic	Yes	[5]
1	Indomethacin	Naproxen	2:1	Melt-quenching	Thermodynamic	Yes	[35]
1	Indomethacin	Naproxen	1:1	Melt-quenching	Thermodynamic	Yes	[35]
1	Indomethacin	Naproxen	1:2	Melt-quenching	Thermodynamic	Yes	[35]
1	Simvastatin	Glipizide	2:1	Ball milling	Kinetic	Yes	[3]
1	Simvastatin	Glipizide	1:1	Ball milling	Kinetic	Yes	[3]
2	Carbamazepine	Arginin	1:1	Ball milling	Kinetic	No	[1]
2	Carbamazepine	Phenylalanin	1:1	Ball milling	Kinetic	No	[1]
2	Carbamazepine	Tryptophan	1:1	Ball milling	Kinetic	Yes	[1]
2	Carbamazepine	Tyrosin	1:1	Ball milling	Kinetic	No	[1]
2	Indomethacin	Arginin	1:1	Ball milling	Kinetic	Yes	[1]
2	Indomethacin	Phenylalanin	1:1	Ball milling	Kinetic	Yes	[1]
2	Indomethacin	Tryptophan	1:1	Ball milling	Kinetic	Yes	[1]
2	Indomethacin	Tyrosin	1:1	Ball milling	Kinetic	No	[1]
3	Olanzapine	Ascorbic acid	1:1	Solvent evaporation	Thermodynamic	Yes	[36]
3	Olanzapine	Ascorbic acid	1:2	Solvent evaporation	Thermodynamic	Yes	[36]
3	Olanzapine	Citric acid	1:1	Solvent evaporation	Thermodynamic	Yes	[36]
3	Olanzapine	Citric acid	1:2	Solvent evaporation	Thermodynamic	Yes	[36]
3	Olanzapine	Tartaric acid	1:1	Solvent evaporation	Thermodynamic	Yes	[36]
3	Olanzapine	Tartaric acid	1:2	Solvent evaporation	Thermodynamic	Yes	[36]
2	Indomethacin	Arginin	1:1	Spray drying	Thermodynamic	Yes	[37]
2	Indomethacin	Arginin	1:1	Ball milling	Kinetic	Yes	[37]
1	Irbesartan	AteNolol	1:1	Hand grinding	Kinetic	Yes	[38]
1	Hydrochlorathiazid	AteNolol	0.4:1	Hand grinding	Kinetic	No	[38]
4	Curcumin	Piperazine	1:2	EtOH-assisted grinding	Kinetic	Yes	[39]
4	Curcumin	Piperazine	1:1	EtOH-assisted grinding	Kinetic	Yes	[39]
3	Loratadine	Citric acid	1:1	Solvent evaporation	Thermodynamic	Yes	[40]
4	Ibuprofen	Nicotinamide	2:1	Solvent evaporation	Thermodynamic	Yes	[41]
4	Ibuprofen	Nicotinamide	1:2	Solvent evaporation	Thermodynamic	Yes	[41]
2	Chloramphenicol	Arginin	1:1	Freez drying	Thermodynamic	No	[42]
2	Chloramphenicol	Cysteine	1:1	Freez drying	Thermodynamic	No	[42]
2	Chloramphenicol	Glycine	1:1	Freez drying	Thermodynamic	No	[42]
2	Chloramphenicol	Leucine	1:1	Freez drying	Thermodynamic	No	[42]
2	Indomethacin	Arginin	1:1	Spray drying	Thermodynamic	Yes	[43]
2	Indomethacin	Arginin	1:2	Spray drying	Thermodynamic	Yes	[43]
2	Ibuprofen	Arginin	1:1	Spray drying	Thermodynamic	Yes	[43]
2	Ibuprofen	Arginin	1:2	Spray drying	Thermodynamic	Yes	[43]
1	Atenolol	Hydrochlorothiazide	1:1	Cryogenic milling	Kinetic	Yes	[44]

1	Atenolol	Hydrochlorothiazide	1:2	Cryogenic milling	Kinetic	Yes	[44]
1	Atenolol	Hydrochlorothiazide	2:1	Cryogenic milling	Kinetic	Yes	[44]
2	Indomethacin	Lysine	1:1	Liquid asissted grinding	Thermodynamic	Yes	[45]
3	Curcumin	Folic acid dihydrate		Liquid asissted grinding	Thermodynamic	Yes	[46]
4	Curcumin	Dextrose	1:1	Liquid asissted grinding	Thermodynamic	No	[46]
3	Curcumin	Suberic acid	1:4	Liquid asissted grinding	Thermodynamic	No	[46]
3	Curcumin	Suberic acid	1:3	Liquid asissted grinding	Thermodynamic	No	[46]
3	Curcumin	Suberic acid	1:2	Liquid asissted grinding	Thermodynamic	No	[46]
3	Curcumin	Suberic acid	1:1	Liquid asissted grinding	Thermodynamic	No	[46]
4	Curcumin	Artemisinin	1:1	Rotavaporization	Thermodynamic	Yes	[47]
2	Simvastatin	Phenylalanin	1:1	Ball milling	Kinetic	Yes	[48]
2	Simvastatin	Tryptophan	1:1	Ball milling	Kinetic	Yes	[48]
2	Simvastatin	Glycine	1:1	Ball milling	Kinetic	No	[48]
2	Simvastatin	Arginin	1:1	Ball milling	Kinetic	No	[48]
2	Simvastatin	Tyrosin	1:1	Ball milling	Kinetic	No	[48]
2	Simvastatin	Leucine	1:1	Ball milling	Kinetic	No	[48]
2	Simvastatin	Cysteine	1:1	Ball milling	Kinetic	No	[48]
2	Carbamazepine	L-Aspartic acid	1:1	Ball milling	Kinetic	No	[48]
2	Carbamazepine	Glutamic acid	1:1	Ball milling	Kinetic	No	[48]
2	Carbamazepine	Glycine	1:1	Ball milling	Kinetic	No	[48]
2	Carbamazepine	Lysine	1:1	Ball milling	Kinetic	No	[48]
2	Carbamazepine	Cysteine	1:1	Ball milling	Kinetic	No	[48]
2	Carbamazepine	Serine	1:1	Ball milling	Kinetic	No	[48]
2	Carbamazepine	Leucine	1:1	Ball milling	Kinetic	No	[48]
2	Furosemide	Arginin	1:1	Ball milling	Kinetic	Yes	[48]
3	Furosemide	L-Aspartic acid	1:1	Ball milling	Kinetic	No	[48]
2	Furosemide	Glycine	1:1	Ball milling	Kinetic	No	[48]
2	Furosemide	Lysine	1:1	Ball milling	Kinetic	Yes	[48]
2	Furosemide	Cysteine	1:1	Ball milling	Kinetic	Yes	[48]
2	Furosemide	Leucine	1:1	Ball milling	Kinetic	Yes	[48]
2	Furosemide	Glutamic acid	1:1	Ball milling	Kinetic	No	[48]
2	Furosemide	Serine	1:1	Ball milling	Kinetic	No	[48]
2	Furosemide	Phenylalanin	1:1	Ball milling	Kinetic	Yes	[48]
2	Indomethacin	Glutamic acid	1:1	Ball milling	Kinetic	No	[48]
2	Indomethacin	L-Aspartic acid	1:1	Ball milling	Kinetic	No	[48]
2	Indomethacin	Glycine	1:1	Ball milling	Kinetic	No	[48]
2	Indomethacin	Lysine	1:1	Ball milling	Kinetic	Yes	[48]
2	Indomethacin	Serine	1:1	Ball milling	Kinetic	No	[48]
2	Indomethacin	Cysteine	1:1	Ball milling	Kinetic	No	[48]
2	Carvedilol	Leucine	1:1	Ball milling	Kinetic	Yes	[48]
2	Carvedilol	Lysine	1:1	Ball milling	Kinetic	No	[48]
2	Carvedilol	Arginin	1:1	Ball milling	Kinetic	No	[48]
2	Carvedilol	Serine	1:1	Ball milling	Kinetic	No	[48]

2	Carvedilol	Tyrosin	1:1	Ball milling	Kinetic	No	[48]
2	Carvedilol	Glutamic acid	1:1	Ball milling	Kinetic	No	[48]
2	Mebendazole	Leucine	1:1	Ball milling	Kinetic	Yes	[48]
2	Mebendazole	Phenylalanin	1:1	Ball milling	Kinetic	Yes	[48]
2	Mebendazole	Glutamic acid	1:1	Ball milling	Kinetic	No	[48]
2	Mebendazole	Cysteine	1:1	Ball milling	Kinetic	No	[48]
2	Mebendazole	Lysine	1:1	Ball milling	Kinetic	Yes	[48]
2	Mebendazole	Glycine	1:1	Ball milling	Kinetic	No	[48]
2	Mebendazole	Arginin	1:1	Ball milling	Kinetic	No	[48]
2	Mebendazole	Tyrosin	1:1	Ball milling	Kinetic	No	[48]
2	Carvedilol	Glycine	1:1	Ball milling	Kinetic	No	[48]
2	Carvedilol	L-Aspartic acid	1:1	Ball milling	Kinetic	No	[48]
2	Carvedilol	Phenylalanin	1:1	Ball milling	Kinetic	Yes	[48]
2	Carvedilol	Cysteine	1:1	Ball milling	Kinetic	No	[48]
2	Indomethacin	Leucine	1:1	Ball milling	Kinetic	Yes	[48]
2	Simvastatin	Glutamic acid	1:1	Ball milling	Kinetic	No	[48]
2	Mebendazole	Tryptophan	1:1	Ball milling	Kinetic	Yes	[48]
2	Mebendazole	L-Aspartic acid	1:1	Ball milling	Kinetic	No	[48]
2	Mebendazole	Serine	1:1	Ball milling	Kinetic	Yes	[48]
4	Atenolol	Urea	1:4	Melt quenching	Thermodynamic	Yes	[49]
3	Carvedilol	Benzoic acid	1,5:1	Spray drying	Thermodynamic	Yes	[50]
3	Carvedilol	Citric acid	2:1	Spray drying	Thermodynamic	Yes	[50]
3	Carvedilol	Malic acid	2:1	Spray drying	Thermodynamic	Yes	[50]
2	Carvedilol	Tryptophan	1:1	Ball milling	Kinetic	Yes	[51]
1	Simvastatin	Nifedipine	1:2	Melt quenching	Thermodynamic	Yes	[52]
1	Simvastatin	Nifedipine	1:1	Melt quenching	Thermodynamic	Yes	[52]
1	Simvastatin	Nifedipine	2:1	Melt quenching	Thermodynamic	Yes	[52]
1	Nifedipine	Paracetamol	1:1,5	Melt quenching	Thermodynamic	Yes	[51]
2	Furosemide	Arginin	1:1,6	Ball milling	Kinetic	Yes	Jensen et al. 2016
4	Olanzapine	Saccharin	1:1	Quench cooling	thermodynamic	Yes	[53]
1	Indomethacin	Paracetamol	1:1	Quench cooling	thermodynamic	Yes	[54]
4	Indomethacin	Nicotinamide	1:1	Quench cooling	thermodynamic	Yes	[54]
2	Quercetin	Arginin	1:2	Ball milling	Kinetic	Yes	[55]
3	Piroxicam	Citric acid	1:1	Spray drying	thermodynamic	Yes	[56]
2	Furosemide	Arginin	1:1	Spray drying	thermodynamic	Yes	[57]
4	Furosemide	Piperine	1:1	Solvent evaporation	thermodynamic	Yes	[57]
1	Telmisartan	Hydrochlorothiacid	1:1	Solvent evaporation	thermodynamic	Yes	[58]
3	Mebendazole	Glutamic acid	1:1	Ball milling	Kinetic	No	[59]
3	Ketokonazole	Fumaric acid	1:1	Neat grinding	Kinetic	Yes	[60]
3	Ketokonazole	Succinic acid	1:1	Neat grinding	Kinetic	No	[60]

*Classification: 1 API-API

2 API – Amino acid

3 API – Organic acid

4 API – Other

** Classification according to Review Liu et al. 2021

Table S2 – List of APIs used in the treatment of COPD and Asthma (Input for Model Application test)

**APIS used in the treatment of asthma and COPD**		
**Inhalable glucocorticoids (IGC)**	**Long-acting muscarinic receptor antagonists (LAMA)**	**Short-acting muscarinic receptor antagonists (SAMA)**
Budesonide	Formoterol	Ipratropium
Fluticasone	Salmeterol	
Beclomethason	Bambuterol	
Ciclesonid		
Mometasone		
**Long-acting beta-adrenoceptor agonist (LABA)**	**Short-acting beta adrenoreceptor agonists (SABA)**	**Additional/Alternative**
Aclidinium	Salbutamol	Montelukast
Glycopyronium	Fenoterol	Chromoglicic acid
Tiotropium	Terbutalin	Theophylline

Table S3– List of APIs used in the treatment of Tuberculosis (Input for Model Application test)

**APIS used in the treatment of tuberculosis**		
**First-line anti-tuberculosis agents**	**Second-line anti-tuberculosis agents**	**Additional/Alternative**
		Fluoroquinolones
Isoniazid	Ethionamide	Ciprofloxacin
Rifampicin	Prothionamide	Ofloxacin
Pyrizinamide	Kanamycin	Levofloxacin
Ethambutol	Amikacin	
Streptomycin	Capreomycin	
	Terizidone	
	Cycloserine	
	Viomycin	
	Para-aminosalicylic acid	

Reference Supplementary Material

[1] K. Löbmann, H. Grohganz, R. Laitinen, C. Strachan, T. Rades, Amino acids as co-amorphous stabilizers for poorly water soluble drugs - Part 1: Preparation, stability and dissolution enhancement, Eur. J. Pharm. Biopharm. 85 (2013) 873–881. https://doi.org/10.1016/j.ejpb.2013.03.014.

[2] K. Löbmann, R. Laitinen, H. Grohganz, C. Strachan, T. Rades, K.C. Gordon, A theoretical and spectroscopic study of co-amorphous naproxen and indomethacin, Int. J. Pharm. 453 (2013) 80–87. https://doi.org/10.1016/j.ijpharm.2012.05.016.

[3] K. Löbmann, C. Strachan, H. Grohganz, T. Rades, O. Korhonen, R. Laitinen, Co-amorphous simvastatin and glipizide combinations show improved physical stability without evidence of intermolecular interactions, Eur. J. Pharm. Biopharm. 81 (2012) 159–169. https://doi.org/10.1016/j.ejpb.2012.02.004.

[4] S. Qian, Z. Li, W. Heng, S. Liang, D. Ma, Y. Gao, J. Zhang, Y. Wei, Charge-assisted intermolecular hydrogen bond formed in coamorphous system is important to relieve the pH-dependent solubility behavior of lurasidone hydrochloride, RSC Adv. 6 (2016) 106396–106412. https://doi.org/10.1039/c6ra18022a.

[5] M. Allesø, N. Chieng, S. Rehder, J. Rantanen, T. Rades, J. Aaltonen, Enhanced dissolution rate and synchronized release of drugs in binary systems through formulation: Amorphous naproxen-cimetidine mixtures prepared by mechanical activation, J. Control. Release. 136 (2009) 45–53.

https://doi.org/10.1016/j.jconrel.2009.01.027.
[6] W. Lu, T. Rades, J. Rantanen, M. Yang, Inhalable co-amorphous budesonide-arginine dry powders prepared by spray drying, Int. J. Pharm. 565 (2019) 1–8. https://doi.org/10.1016/j.ijpharm.2019.04.036.
[7] M. Zhang, X. Xiong, Z. Suo, Q. Hou, N. Gan, P. Tang, X. Ding, H. Li, Co-amorphous palbociclib-organic acid systems with increased dissolution rate, enhanced physical stability and equivalent biosafety, RSC Adv. 9 (2019) 3946–3955. https://doi.org/10.1039/c8ra09710k.
[8] R. Mizoguchi, H. Waraya, Y. Hirakura, Application of Co-Amorphous Technology for Improving the Physicochemical Properties of Amorphous Formulations, Mol. Pharm. 16 (2019) 2142–2152. https://doi.org/10.1021/acs.molpharmaceut.9b00105.
[9] S. Yamamura, H. Gotoh, Y. Sakamoto, Y. Momose, Physicochemical properties of amorphous precipitates of cimetidine-indomethacin binary system, Eur. J. Pharm. Biopharm. 49 (2000) 259–265. https://doi.org/10.1016/S0939-6411(00)00060-6.
[10] S.J. Dengale, O.P. Ranjan, S.S. Hussen, B.S.M. Krishna, P.B. Musmade, G. Gautham Shenoy, K. Bhat, Preparation and characterization of co-amorphous Ritonavir-Indomethacin systems by solvent evaporation technique: Improved dissolution behavior and physical stability without evidence of intermolecular interactions, Eur. J. Pharm. Sci. 62 (2014) 57–64. https://doi.org/10.1016/j.ejps.2014.05.015.
[11] A. Shayanfar, H. Ghavimi, H. Hamishehkar, A. Jouyban, Coamorphous atorvastatin calcium to improve its physicochemical and pharmacokinetic properties, J. Pharm. Pharm. Sci. 16 (2013) 577–587. https://doi.org/10.18433/j3xs4s.
[12] J. Knapik, Z. Wojnarowska, K. Grzybowska, K. Jurkiewicz, L. Tajber, M. Paluch, Molecular Dynamics and Physical Stability of Coamorphous Ezetimib and Indapamide Mixtures, Mol. Pharm. 12 (2015) 3610–3619. https://doi.org/10.1021/acs.molpharmaceut.5b00334.
[13] S. Wairkar, R. Gaud, Co-Amorphous Combination of Nateglinide-Metformin Hydrochloride for Dissolution Enhancement, AAPS PharmSciTech. 17 (2016) 673–681. https://doi.org/10.1208/s12249-015-0371-4.
[14] A. Teja, P.B. Musmade, A.B. Khade, S.J. Dengale, Simultaneous improvement of solubility and permeability by fabricating binary glassy materials of Talinolol with Naringin: Solid state characterization, in-vivo in-situ evaluation, Eur. J. Pharm. Sci. 78 (2015) 234–244. https://doi.org/10.1016/j.ejps.2015.08.002.
[15] Renuka, S.K. Singh, M. Gulati, R. Narang, Stable amorphous binary systems of glipizide and atorvastatin powders with enhanced dissolution profiles: formulation and characterization, Pharm. Dev. Technol. 22 (2017) 13–25. https://doi.org/10.3109/10837450.2015.1125921.
[16] A. Beyer, L. Radi, H. Grohganz, K. Löbmann, T. Rades, C.S. Leopold, Preparation and recrystallization behavior of spray-dried co-amorphous naproxen-indomethacin, Eur. J. Pharm. Biopharm. 104 (2016) 72–81. https://doi.org/10.1016/j.ejpb.2016.04.019.
[17] M.G. Russo, M.I. Sancho, L.M.A. Silva, H.A. Baldoni, T. Venancio, J. Ellena, G.E. Narda, Looking for the interactions between omeprazole and amoxicillin in a disordered phase. An experimental and theoretical study, Spectrochim. Acta - Part A Mol. Biomol. Spectrosc. 156 (2016) 70–77. https://doi.org/10.1016/j.saa.2015.11.021.
[18] H. Ueda, N. Muranushi, S. Sakuma, Y. Ida, T. Endoh, K. Kadota, Y. Tozuka, A Strategy for Co-former Selection to Design Stable Co-amorphous Formations Based on Physicochemical Properties of Non-steroidal Inflammatory Drugs, Pharm. Res. 33 (2016) 1018–1029. https://doi.org/10.1007/s11095-015-1848-2.
[19] A.W. Lim, K. Löbmann, H. Grohganz, T. Rades, N. Chieng, Investigation of physical properties and stability of indomethacin-cimetidine and naproxen-cimetidine co-amorphous systems prepared by quench cooling, coprecipitation and ball milling, J. Pharm. Pharmacol. 68 (2016) 36–45. https://doi.org/10.1111/jphp.12494.
[20] Y. Gao, J. Liao, X. Qi, J. Zhang, Coamorphous repaglinide-saccharin with enhanced dissolution, Int. J. Pharm. 450 (2013) 290–295. https://doi.org/10.1016/j.ijpharm.2013.04.032.
[21] S. Qian, W. Heng, Y. Wei, J. Zhang, Y. Gao, Coamorphous lurasidone hydrochloride-saccharin with charge-assisted hydrogen bonding interaction shows improved physical stability and enhanced dissolution with ph-independent solubility behavior, Cryst. Growth Des. 15 (2015) 2920–2928. https://doi.org/10.1021/acs.cgd.5b00349.
[22] K.T. Jensen, K. Löbmann, T. Rades, H. Grohganz, Improving co-amorphous drug formulations by the addition of the highly water soluble amino acid, Proline, Pharmaceutics. 6 (2014) 416–435. https://doi.org/10.3390/pharmaceutics6030416.
[23] R. Laitinen, K. Löbmann, H. Grohganz, C. Strachan, T. Rades, Amino acids as Co-amorphous excipients for simvastatin and glibenclamide: Physical properties and stability, Mol. Pharm. 11 (2014) 2381–2389.

https://doi.org/10.1021/mp500107s.
[24] E. Lenz, K.T. Jensen, L.I. Blaabjerg, K. Knop, H. Grohganz, K. Löbmann, T. Rades, P. Kleinebudde, Solid-state properties and dissolution behaviour of tablets containing co-amorphous indomethacin-arginine., Eur. J. Pharm. Biopharm. 96 (2015) 44–52. https://doi.org/10.1016/j.ejpb.2015.07.011.
[25] K.T. Jensen, F.H. Larsen, C. Cornett, K. Löbmann, H. Grohganz, T. Rades, Formation Mechanism of Coamorphous Drug-Amino Acid Mixtures, Mol. Pharm. 12 (2015) 2484–2492. https://doi.org/10.1021/acs.molpharmaceut.5b00295.
[26] G. Craye, K. Löbmann, H. Grohganz, T. Rades, R. Laitinen, Characterization of amorphous and co-amorphous simvastatin formulations prepared by spray drying, Molecules. 20 (2015) 21532–21548. https://doi.org/10.3390/molecules201219784.
[27] P. Hoppu, K. Jouppila, J. Rantanen, S. Schantz, A.M. Juppo, Characterisation of blends of paracetamol and citric acid, J. Pharm. Pharmacol. 59 (2010) 373–381. https://doi.org/10.1211/jpp.59.3.0006.
[28] A.M.A. Ali, A.A. Ali, I.A. Maghrabi, Clozapine-carboxylic acid plasticized co-amorphous dispersions: Preparation, characterization and solution stability evaluation, Acta Pharm. 65 (2015) 133–146. https://doi.org/10.1515/acph-2015-0014.
[29] Y. Han, Y. Pan, J. Lv, W. Guo, J. Wang, Powder grinding preparation of co-amorphous β-azelnidipine and maleic acid combination: Molecular interactions and physicochemical properties, Powder Technol. 291 (2016) 110–120. https://doi.org/10.1016/j.powtec.2015.11.068.
[30] T. Masuda, Y. Yoshihashi, E. Yonemochi, K. Fujii, H. Uekusa, K. Terada, Cocrystallization and amorphization induced by drug-excipient interaction improves the physical properties of acyclovir, Int. J. Pharm. 422 (2012) 160–169. https://doi.org/10.1016/j.ijpharm.2011.10.046.
[31] Y. Huang, Q. Zhang, J.R. Wang, K.L. Lin, X. Mei, Amino acids as co-amorphous excipients for tackling the poor aqueous solubility of valsartan, Pharm. Dev. Technol. 22 (2017) 69–76. https://doi.org/10.3109/10837450.2016.1163390.
[32] Q. Lu, G. Zografi, Phase Behavior of Binary and Ternary Amorphous Mixtures Containing Indomethacin, Citric Acid and PVP, Pharm. Res. 15 (1998) 1202-.
[33] K. Gniado, K. Löbmann, T. Rades, A. Erxleben, The influence of co-formers on the dissolution rates of co-amorphous sulfamerazine/excipient systems, Int. J. Pharm. 504 (2016) 20–26. https://doi.org/10.1016/j.ijpharm.2016.03.023.
[34] Q. Hu, D.S. Choi, H. Chokshi, N. Shah, H. Sandhu, Highly efficient miniaturized coprecipitation screening (MiCoS) for amorphous solid dispersion formulation development, Int. J. Pharm. 450 (2013) 53–62. https://doi.org/10.1016/j.ijpharm.2013.04.040.
[35] K. Löbmann, R. Laitinen, H. Grohganz, K.C. Gordon, C. Strachan, T. Rades, Coamorphous drug systems: Enhanced physical stability and dissolution rate of indomethacin and naproxen, Mol. Pharm. 8 (2011) 1919–1928. https://doi.org/10.1021/mp2002973.
[36] E.M. Maher, A.M.A. Ali, H.F. Salem, A.A. Abdelrahman, In vitro/in vivo evaluation of an optimized fast dissolving oral film containing olanzapine co-amorphous dispersion with selected carboxylic acids, Drug Deliv. 23 (2016) 3088–3100. https://doi.org/10.3109/10717544.2016.1153746.
[37] K.T. Jensen, L.I. Blaabjerg, E. Lenz, A. Bohr, H. Grohganz, P. Kleinebudde, T. Rades, K. Löbmann, Preparation and characterization of spray-dried co-amorphous drug-amino acid salts, J. Pharm. Pharmacol. 68 (2015) 615–624. https://doi.org/10.1111/jphp.12458.
[38] J. Haneef, R. Chadha, Drug-Drug Multicomponent Solid Forms: Cocrystal, Coamorphous and Eutectic of Three Poorly Soluble Antihypertensive Drugs Using Mechanochemical Approach, AAPS PharmSciTech. 18 (2017) 2279–2290. https://doi.org/10.1208/s12249-016-0701-1.
[39] W. Pang, J. Lv, S. Du, J. Wang, J. Wang, Y. Zeng, Preparation of Curcumin-Piperazine Coamorphous Phase and Fluorescence Spectroscopic and Density Functional Theory Simulation Studies on the Interaction with Bovine Serum Albumin, Mol. Pharm. 14 (2017) 3013–3024. https://doi.org/10.1021/acs.molpharmaceut.7b00217.
[40] J. Wang, R. Chang, Y. Zhao, J. Zhang, T. Zhang, Q. Fu, C. Chang, A. Zeng, Coamorphous Loratadine-Citric Acid System with Enhanced Physical Stability and Bioavailability, AAPS PharmSciTech. 18 (2017) 2541–2550. https://doi.org/10.1208/s12249-017-0734-0.
[41] Y. Bi, D. Xiao, S. Ren, S. Bi, J. Wang, F. Li, The Binary System of Ibuprofen-Nicotinamide Under Nanoscale Confinement: From Cocrystal to Coamorphous State, J. Pharm. Sci. 106 (2017) 3150–3155. https://doi.org/10.1016/j.xphs.2017.06.005.
[42] V.B. Sterren, V. Aiassa, C. Garnero, Y.G. Linck, A.K. Chattah, G.A. Monti, M.R. Longhi, A. Zoppi, Preparation of Chloramphenicol/Amino Acid Combinations Exhibiting Enhanced Dissolution Rates and Reduced Drug-Induced Oxidative Stress, AAPS PharmSciTech. 18 (2017) 2910–2918. https://doi.org/10.1208/s12249-017-0775-4.

[43] R. Ojarinta, L. Lerminiaux, R. Laitinen, Spray drying of poorly soluble drugs from aqueous arginine solution, Int. J. Pharm. 532 (2017) 289–298. https://doi.org/10.1016/j.ijpharm.2017.09.015.

[44] S.M. Moinuddin, S. Ruan, Y. Huang, Q. Gao, Q. Shi, B. Cai, T. Cai, Facile formation of co-amorphous atenolol and hydrochlorothiazide mixtures via cryogenic-milling: Enhanced physical stability, dissolution and pharmacokinetic profile, Int. J. Pharm. 532 (2017) 393–400. https://doi.org/10.1016/j.ijpharm.2017.09.020.

[45] G. Kasten, K. Nouri, H. Grohganz, T. Rades, K. Löbmann, Performance comparison between crystalline and co-amorphous salts of indomethacin-lysine, Int. J. Pharm. 533 (2017) 138–144. https://doi.org/10.1016/j.ijpharm.2017.09.063.

[46] J.M. Skieneh, I. Sathisaran, S.V. Dalvi, S. Rohani, Co-amorphous form of curcumin-folic acid dihydrate with increased dissolution rate, Cryst. Growth Des. 17 (2017) 6273–6280. https://doi.org/10.1021/acs.cgd.7b00947.

[47] K. Suresh, M.K.C. Chaitanya Mannava, A. Nangia, A novel curcumin-artemisinin coamorphous solid: Physical properties and pharmacokinetic profile, RSC Adv. 4 (2014) 58357–58361. https://doi.org/10.1039/c4ra11935e.

[48] G. Kasten, H. Grohganz, T. Rades, K. Löbmann, Development of a screening method for co-amorphous formulations of drugs and amino acids, Eur. J. Pharm. Sci. 95 (2016) 28–35. https://doi.org/10.1016/j.ejps.2016.08.022.

[49] Y. Hirakawa, H. Ueda, T. Miyano, N. Kamiya, M. Goto, New insight into transdermal drug delivery with supersaturated formulation based on co-amorphous system, Int. J. Pharm. 569 (2019) 118582. https://doi.org/10.1016/j.ijpharm.2019.118582.

[50] W. Wu, H. Ueda, K. Löbmann, T. Rades, H. Grohganz, Organic acids as co-formers for co-amorphous systems – Influence of variation in molar ratio on the physicochemical properties of the co-amorphous systems, Eur. J. Pharm. Biopharm. 131 (2018) 25–32. https://doi.org/10.1016/j.ejpb.2018.07.016.

[51] E.O. Kissi, K. Khorami, T. Rades, Determination of stable co-amorphous drug–drug ratios from the eutectic behavior of crystalline physical mixtures, Pharmaceutics. 11 (2019). https://doi.org/10.3390/pharmaceutics11120628.

[52] C. Martínez-Jiménez, J. Cruz-Angeles, M. Videa, L.M. Martínez, Co-amorphous simvastatin-nifedipine with enhanced solubility for possible use in combination therapy of hypertension and hypercholesterolemia, Molecules. 23 (2018) 1–13. https://doi.org/10.3390/molecules23092161.

[53] N.F. da Costa, A.I. Fernandes, J.F. Pinto, Measurement of the amorphous fraction of olanzapine incorporated in a co-amorphous formulation, Int. J. Pharm. 588 (2020) 119716. https://doi.org/10.1016/j.ijpharm.2020.119716.

[54] H. Fael, A.L. Demirel, Indomethacin co-amorphous drug-drug systems with improved solubility, supersaturation, dissolution rate and physical stability, Int. J. Pharm. 600 (2021) 120448. https://doi.org/10.1016/j.ijpharm.2021.120448.

[55] P. Hatwar, I.B. Pathan, N.A.H. Chishti, W. Ambekar, Pellets containing quercetin amino acid co-amorphous mixture for the treatment of pain: Formulation, optimization, in-vitro and in-vivo study, J. Drug Deliv. Sci. Technol. 62 (2021) 102350. https://doi.org/10.1016/j.jddst.2021.102350.

[56] Y. Hirakawa, H. Ueda, Y. Takata, K. Minamihata, R. Wakabayashi, N. Kamiya, M. Goto, Co-amorphous formation of piroxicam-citric acid to generate supersaturation and improve skin permeation, Eur. J. Pharm. Sci. 158 (2021) 105667. https://doi.org/10.1016/j.ejps.2020.105667.

[57] M. Ruponen, K. Kettunen, M.S. Pires, R. Laitinen, Co-amorphous formulations of furosemide with arginine and p-glycoprotein inhibitor drugs, Pharmaceutics. 13 (2021) 1–18. https://doi.org/10.3390/pharmaceutics13020171.

[58] X. Shi, X. Zhou, S. Shen, Q. Chen, S. Song, C. Gu, C. Wang, Improved in vitro and in vivo properties of telmisartan in the co-amorphous system with hydrochlorothiazide: A potential drug-drug interaction mechanism prediction, Eur. J. Pharm. Sci. 161 (2021) 105773. https://doi.org/10.1016/j.ejps.2021.105773.

[59] W. Wu, H. Grohganz, T. Rades, K. Löbmann, Comparison of co-former performance in co-amorphous formulations: Single amino acids, amino acid physical mixtures, amino acid salts and dipeptides as co-formers, Eur. J. Pharm. Sci. 156 (2021) 105582. https://doi.org/10.1016/j.ejps.2020.105582.

[60] M.S. Hossain Mithu, S.A. Ross, A.P. Hurt, D. Douroumis, Effect of mechanochemical grinding conditions on the formation of pharmaceutical cocrystals and co-amorphous solid forms of ketoconazole – Dicarboxylic acid, J. Drug Deliv. Sci. Technol. 63 (2021) 102508. https://doi.org/10.1016/j.jddst.2021.102508.
